# Long non-coding RNA CACNA1G-AS1 promotes proliferation and invasion and inhibits apoptosis by regulating expression of miR-205 in human keloid fibroblasts

**DOI:** 10.1042/BSR20192839

**Published:** 2020-06-18

**Authors:** Xu Zhao, Xiang Jie, Ya-kun Gao, Bing Nie, Hua Jiang

**Affiliations:** Department of Plastic and Reconstructive Surgery, Changzheng Hospital, Second Military Medical University, Shanghai 200003, China

**Keywords:** apoptosis, CACNA1G-AS1, invasion, keloid fibroblasts, miR-205, proliferation

## Abstract

**Background:** Keloid is a fibrous tissue proliferative disease in which proliferative scars grow beyond the boundary of the original wound skin. Long non-coding RNAs (lncRNAs), as competing endogenous RNAs (ceRNAs), bind to microRNAs (miRNAs) to regulate various biological processes. The present study was aim to illuminate the mechanism of calcium voltage-gated channel subunit alpha1 G antisense RNA 1 (CACNA1G-AS1) in human keloid fibroblasts. **Methods:** CACNA1G-AS1 and miR-205 levels were detected using quantitative real-time polymerase chain reaction (qRT-PCR). Cell Counting Kit-8 (CCK-8) assay was used to measure the proliferation and transwell assay was performed to evaluate cell invasion. Furthermore, the apoptosis rates of cells were evaluated by flow cytometry analysis, and the activity of caspase-3 in keloid fibroblasts was tested by Caspase-3 activity assay. Dual luciferase reporter assay was carried out to examine the relationship between CACNA1G-AS1 and miR-205 and RNA immunoprecipitation (RIP) assay was conducted to further confirm the relation. **Results:** CACNA1G-AS1 level was up-regulated in keloid tissues and keloid fibroblasts. CACNA1G-AS1 overexpression promoted proliferation and invasion and suppressed apoptosis of keloid fibroblasts. Moreover, miR-205 was targeted by CACNA1G-AS1 and miR-205 was markedly decreased in keloid tissues and keloid fibroblasts. Also, miR-205 expression was negatively regulated by CACNA1G-AS1 and miR-205 silencing enhanced proliferation and invasion and inhibited apoptosis. Furthermore, CACNA1G-AS1 and miR-205 played the antagonistic role in miR-205 expression, proliferation, invasion, and apoptosis of keloid fibroblasts. **Conclusion:** CACNA1G-AS1 suppressed miR-205 expression to promote proliferation and invasion and inhibit apoptosis in human keloid fibroblasts.

## Introduction

Keloid, which is also named as keloid disorder or keloidal scar, is a pathologic fibro-proliferative disease caused by excessive wound healing after skin tissue injury, involving excessive growth beyond the boundary of the original wound skin, invasion of nearby normal tissues, non-degenerative changes, and recurrent growth [1,2]. Also, keloid can lead to the loss of biological and physical function in the wound skin. Nowadays, more than half of patients suffered from keloid in the world, resulting in a decline in quality of life and physical and psychological pain [3]. It is widely reported that pathological scar formation is caused by abnormal proliferation of fibroblasts [4]. Although numerous studies have provided some evidence to illuminate the progress, the molecular mechanism underlying keloid formation needs to be further explored. Hence, better understanding of keloid is extremely necessary for discovering a novel and effective treatment for keloid.

Long non-coding RNAs (lncRNAs) are now identified as a class of newly discovered transcripts that contain more than 200 nucleotides in length [5,6]. More and more studies showed that lncRNAs play an important role in different biological processes, such as cell growth, proliferation, and apoptosis [7]. It has been proposed that plenty of lncRNAs were closely related to the progression of keloid fibrosis [8], such as HNF1A-AS1 [9], COL1A2-AS1 [10], CACNA1G-AS1, HOXA11-AS, LINC00312, and RP11-91I11.1 [11]. Recent study demonstrated that calcium voltage-gated channel subunit alpha1 G antisense RNA 1 (CACNA1G-AS1) was up-regulated in keloid and had a positive effect on cell migration in keloid fibrosis [12]. However, the expression and function of lncRNA CACNA1G-AS1 remain unclear in human keloid fibroblasts up to date.

MicroRNAs (miRNAs), noncoding small RNAs with approximately 22 nucleotides in length, played important roles in tumorigenesis and progression [13,14]. Numerous researches focus on miRNA roles as an essential epigenetic regulator in many biological processes, including carcinogenesis and fibroblast activation [15,16]. Previously, some miRNAs have been proved to be associated with cell growth, proliferation and differentiation of keloid fibroblasts. For example, miR-188-5p and miR-203 inhibited the proliferation and invasion of keloid fibroblasts [17,18]. It has been demonstrated that MiR-196a knockdown enhanced type I and type III collagen expression in keloid fibroblasts [19]. Wang et al. demonstrated that miR-21 was highly expressed in keloid and regulated the progression of human keloid fibroblasts [20]. Zhang et al. showed that miR-153-5p was identified to play an anti-fibrotic role and suppress the progression of human keloid fibroblasts [21]. A previous study reported that miR-205 was down-regulated in keloid tissues, and overexpression of miR-205 suppressed the progression of keloid [22]. However, there is no relevant study on the interaction between CACNA1G-AS1 and miR-205 in keloid fibroblasts.

In the present study, we explored the relationship between CACNA1G-AS1 and miR-205 and further studied their effects on proliferation, invasion, and apoptosis of keloid fibroblasts.

## Materials and methods

### Acquisition of tissue samples

Paired human keloid tissues and adjacent normal tissues were collected from patients with keloid in the Changzheng Hospital, Second Military Medical University in 2018. Each patient provided with the informed consent prior to surgery. All experiments in the present study were approved by the Changzheng Hospital, Second Military Medical University Ethical Committee. The research has been carried out in accordance with the World Medical Association Declaration of Helsinki, and that all subjects provided written informed consent.

### Primary fibroblast isolation and cell culture

Normal fibroblasts (NF) and primary keloid fibroblasts (KF) were original from normal skin and keloid skin tissues (*n* = 3; random selected three groups) collected from keloid patients. After keloid tissues were washed with phosphate-buffered saline (PBS) solution for two or three times, epidermis and subcutaneous adipose tissue were removed from keloid tissues. Then, specimens were shredded and digested with trypsin. After filtration and centrifugation, cells were incubated at 37°C in Dulbecco’s modified Eagle medium (DMEM, Gibco, Carlsbad, CA, U.S.A.) along with 10% fetal bovine serum (FBS, Hyclone, Logan, UT, U.S.A.).

### Cell transfection

The CACNA1G-AS1 and its control (pcDNA), small interference RNA (siRNA) targeting CACNA1G-AS1 (si-CACNA1G-AS1) and its control (si-NC), miR-205 and its matched control (miR-NC), anti-miR-205 and its corresponding control (anti-miR-NC) were chemically synthesized by Sangon biotech (Shanghai, China). Keloid fibroblasts transfection was performed by using Lipofectamine 2000 (Invitrogen, Carlsbad, CA, U.S.A.).

### Quantitative real-time polymerase chain reaction

Total RNA of keloid fibroblasts was extracted by using RNApure total RNA fast isolation kit (TransGen Biotech, Beijing, China) according to the reference books. The first strand of cDNA was amplified by PCR machine using PrimeScript reverse transcriptase reagent kit (Takara, Dalian, China). MiScript reverse transcription kit (Qiagen, Hilden, Germany) was selected to perform the miRNA reverse transcription. Quantitative real-time polymerase chain reaction (qRT-PCR) assay was performed by using iQ™ SYBR® Green Supermix (Bio-Rad Laboratories, Philadelphia, PA, U.S.A.) with the following primers: CACNA1G-AS1 (forward, 5′-TGTGCTTCACCATGCTCCAT-3′; reverse, 5′-ATTAGTGCTCCGGCCAACAA-3′), miR-205 (forward, 5′-CTTGTCCTTCATTCCACCGGA-3′; reverse, 5′-TGCCGCCTGAACTTCACTCC-3′), GAPDH (forward, 5′-TGTTCGTCATGGGTGTGAAC-3′; reverse, 5′-ATGGCATGGACTGTGGTCAT-3′), U6: (forward, 5′-CTCGCTTCGGCAGCACA-3′; reverse, 5′-AACGCTTCACGAATTTGCGT-3′). After amplification, the results were normalized by the formula: $2^{-\text{ΔΔ}C_{\text{t}}}$, with GAPDH or U6 as internal control.

### Cell Counting Kit-8 assay

The proliferation of transfected cells was evaluated by Cell Counting Kit-8 (CCK-8). Keloid fibroblasts and normal skin cells were placed into plates. After being cultivated in incubator for 48 h, keloid cells were interacted with CCK-8 solution at 37°C. At last, the absorbance at 450 nm of transfected cells wasasured by a microplate reader (Bio-Rad Laboratories).

### Transwell assay

Cell invasion was detected by transwell method. Keloid fibroblasts were incubated for 24 h and were added to the upper chambers (Corning, New York, NY, U.S.A.) which were pre-coated with Matrigel (BD Biosciences, Sparks, MD, U.S.A.). In addition, the lower chambers were filed with DMEM medium containing 10% FBS. The keloid fibroblasts that had invaded into the lower chamber were collected and fixed with methanol and stained with 0.5% crystal violet (Sigma, St. Louis, MO, U.S.A.). Finally, the number of invaded cells was counted by a Countess automatic cell counter (Invitrogen) with three random views.

### Flow cytometry assay

Keloid fibroblasts were placed into six-well plates. After washing with PBS twice, keloid fibroblasts were resuspended in binding buffer. The ratio of apoptotic keloid fibroblasts was evaluated by the Annexin V-fluorescein isothiocyanate (FITC) Apoptosis Detection Kit (Thermo Fisher Scientific, Rockford, IL, U.S.A.). At last, the apoptosis was assessed by FACSCalibur flow cytometer (BD Biosciences).

### Caspase-3 activity assay

The activity of caspase-3 was evaluated by the caspase activity kit referring to the use manual. Briefly, after incubation for 24 h, cells were collected and treated with ice-cold radioimmunoprecipitation assay buffer (RIPA, Beyotime, Shanghai, China). Then, cell suspension was centrifuged and washed. The concentration of extracted protein was determined by bicinchoninic acid (BCA) kit. Finally, the activity of caspase-3 were measured by microplate reader.

### Dual luciferase reporter assay

Partial sequences of the wild type (wt) or mutant (mut) of CACNA1G-AS1 were synthesized and constructed into the psiCHECK-2 luciferase vector (Promega, Madison, WI, U.S.A.). The mutant sequences of CACNA1G-AS1 were synthesized from Sangon Biotech (Sangon). Next, these luciferase reporter plasmids were transfected into keloid fibroblasts using Lipofectamine 2000 transfection reagent (Invitrogen). After cultivation in incubator for 48 h, cells were harvested. Luciferase activity was examined by Dual Luciferase Reporter Assay Kit (Promega) to confirm the relation between CACNA1G-AS1 and miR-205.

### RNA immunoprecipitation assay

Keloid fibroblasts were transfected with miR-NC or miR-205, respectively. Then, cell lysates were harvested and were added with magnetic beads. Next, magnetic beads were collected and then re-suspended in Wash Buffer. RNA immunoprecipitation (RIP) assays were performed using the Magna RIPTM RNA Binding Protein Immunoprecipitation Kit (Millipore). Finally, keloid fibroblasts were incubated with antibody against RIP-Ago2 (Millipore) or IgG (Millipore). QRT-PCR assay was conducted to measure the enrichment of CACNA1G-AS1 in transfected keloid fibroblasts.

### Statistical analysis

All above data were shown as mean ± standard deviation (SD) from at least three independent experiments. The comparisons between two groups were estimated by Student’s *t*-test method and one-way analysis of variance (ANOVA) was performed to compare the differences among at least three groups. *P*<0.01 and *P*<0.001 were all considered as statistically significant difference.

## Results

### CACNA1G-AS1 was highly expressed in keloid tissues and keloid fibroblasts

**Figure 1:**
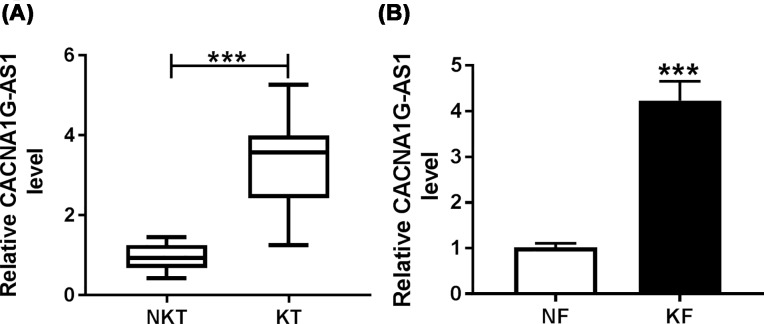
Expression level of CACNA1G-AS1 was up-regulated in keloid tissues and keloid fibroblasts (**A,B**) The expression of CACNA1G-AS1 was detected in normal skin tissues (NKT), keloid tissues (KT), normal fibroblasts (NF), and keloid fibroblasts (KF) by qRT-PCR assay. ***P<0.001.

First, qRT-PCR was conducted to detect the expression level of CACNA1G-AS1 in normal skin tissues, keloid tissues, normal fibroblasts, and keloid fibroblasts. Compared with the normal skin tissues, CACNA1G-AS1 expression was noticeably elevated in keloid tissues (*n*=20) (Figure 1A). Additionally, the level of CACNA1G-AS1 in keloid fibroblasts was significantly increased versus normal fibroblasts (Figure 1B). These results indicated that CACNA1G-AS1 might play a role in the development of human keloid fibroblasts.

### CACNA1G-AS1 regulated proliferation, invasion, and apoptosis of keloid fibroblasts

**Figure 2:**
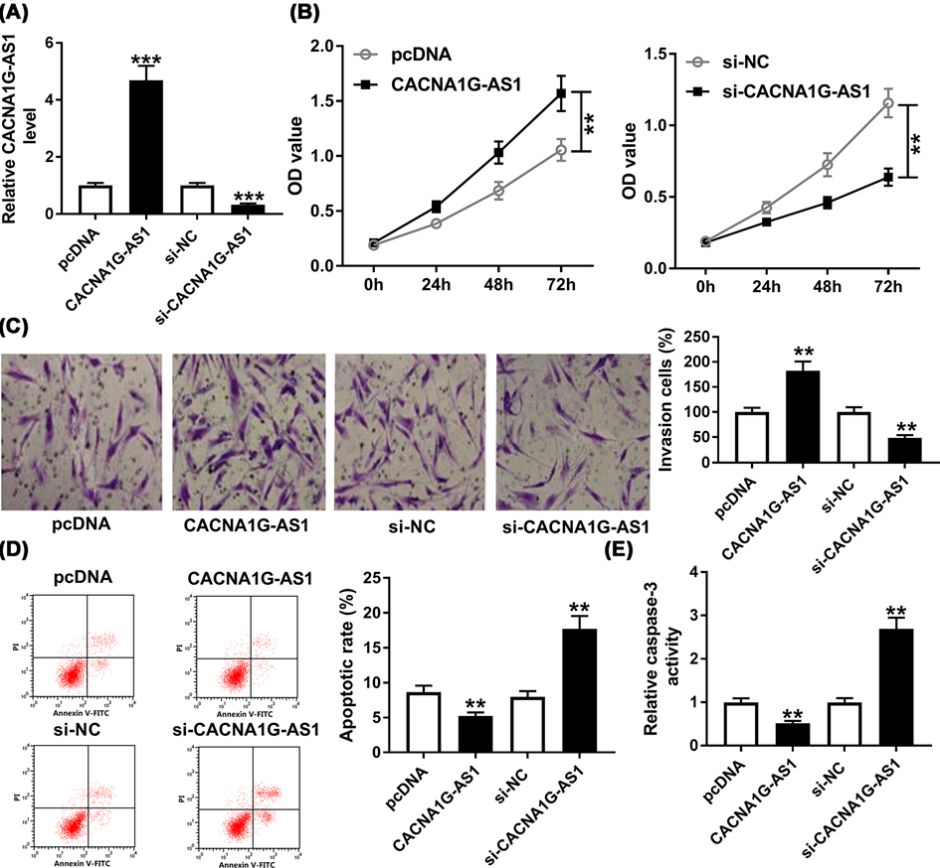
CACNA1G-AS1 knockdown repressed proliferation and invasion and promoted apoptosis of keloid fibroblasts Keloid fibroblasts were transfected with pcDNA, CACNA1G-AS1, si-NC, or si-CACNA1G-AS1. (**A**) The expression of CACNA1G-AS1 was measured by qRT-PCR assay in transfected keloid fibroblasts. (**B**) Cell viability was assessed by CCK-8 assay in transfected keloid fibroblasts. (**C**) The invasion of transfected keloid fibroblasts was evaluated by transwell assay. (**D**) Flow cytometry assay was used to detect the apoptosis rate of transfected keloid fibroblasts. (**E**) The activity of caspase-3 in transfected keloid fibroblasts was measured by Caspase-3 activity assay. **P<0.01, ***P<0.001.

To examine the role of CACNA1G-AS1 in keloid fibroblasts proliferation, invasion, and apoptosis, the keloid fibroblasts were transfected with pcDNA, CACNA1G-AS1, si-NC, or si-CACNA1G-AS1. QRT-PCR assay showed that CACNA1G-AS1 expression was greatly increased by CACNA1G-AS1 and was sharply inhibited by the interference of CACNA1G-AS1 (Figure 2A). Then the results of CCK-8 assay suggested that overexpression of CACNA1G-AS1 notably promoted the proliferation of keloid fibroblasts and the silence of CACNA1G-AS1 had the opposite effect on proliferation with CACNA1G-AS1 overexpression (Figure 2B). Besides, transwell assay demonstrated that the number of invaded keloid fibroblasts was significantly increased in CACNA1G-AS1 transfected keloid fibroblasts, which was decreased by CACNA1G-AS1 silencing (Figure 2C). Moreover, the apoptosis of keloid fibroblasts was markedly inhibited by CACNA1G-AS1 overexpression and was promoted by the transfection with si-CACNA1G-AS1 (Figure 2D). The protein related to apoptosis was detected by Caspase-3 activity assay. The result revealed that CACNA1G-AS1 significantly reduced caspase-3 activity and silenced CACNA1G-AS1 promoted caspase-3 activity (Figure 2E). These data revealed that CACNA1G-AS1 participated in the regulation of proliferation, invasion, and apoptosis of keloid fibroblasts *in vitro*.

### CACNA1G-AS1 directly interacted with miR-205

**Figure 3:**
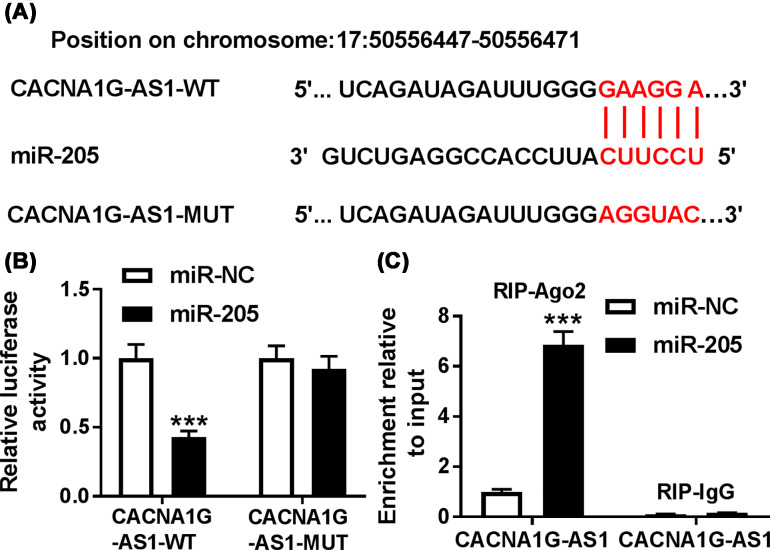
CACNA1G-AS1 could bind to miR-205 specifically (**A**) The putative binding sites between CACNA1G-AS1 and miR-205 and the mutant sequences of CACNA1G-AS1 were shown. (**B**) Relative luciferase activity was measured in keloid fibroblasts co-transfected with PART1-WT or PART1-MUT and miR-205 or miR-NC. (**C**) RIP assay was performed to examine the enrichment of CACNA1G-AS1 in keloid fibroblasts co-transfected with miR-205 or miR-NC and RIP-AGO2 or RIP-IgG. ***P<0.001.

Next, the potential targets of CACNA1G-AS1 were predicted using LncBase Predicted v.2. The putative binding sites between CACNA1G-AS1 and miR-205 and the mutant sequences of CACNA1G-AS1 were shown in Figure 3A. Subsequently, to determine the interaction between them, luciferase reporter vectors were co-transfected into keloid fibroblasts with miR-205 or miR-NC. Luciferase activities of keloid fibroblasts in CACNA1G-AS1-WT group was significantly decreased by the transfection of miR-205, while the activities of mutated group remained unchanged (Figure 3B). Moreover, RIP assay indicated that the introduction of miR-205 resulted in the significant enrichment of CACNA1G-AS1 in RIP-AGO2 complex compared with RIP-IgG control (Figure 3C). All above data implied that miR-205 was targeted by CACNA1G-AS1.

### Expression level of miR-205 was drastically decreased in keloid tissues and keloid fibroblasts and was negatively regulated by CACNA1G-AS1

**Figure 4:**
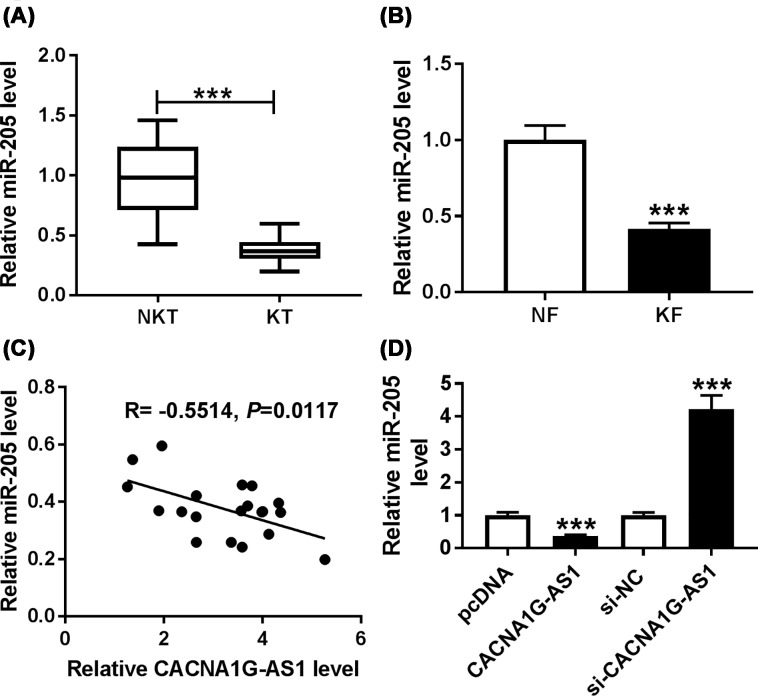
The expression of miR-205 was up-regulated in keloid tissues and keloid fibroblasts and miR-205 expression was down-regulated by CACNA1G-AS1 (**A,B**) QRT-PCR was carried out to detect the expression of miR-205 in normal skin tissues (NKT), keloid tissues (KT), normal fibroblasts (NF), and keloid fibroblasts (KF). (**C**) Correlation analysis between CACNA1G-AS1 and miR-205 expression in keloid fibroblasts was performed by Pearson analysis. (**D**) The expression of miR-205 was measured by qRT-PCR in keloid fibroblasts transfected with pcDNA, CACNA1G-AS1, si-NC, or si-CACNA1G-AS1. ***P<0.001.

Then, qRT-PCR assay demonstrated that the expression of miR-205 was specifically lower in keloids tissues than in the normal skin tissues (Figure 4A). In addition, compared with human normal fibroblasts, the expression of miR-205 in keloid fibroblasts was down-regulated (Figure 4B). Furthermore, the correlation analysis disclosed that there was an opposite trend between CACNA1G-AS1 and miR-205 expression (Figure 4C). To explore the interaction between CACNA1G-AS1 and miR-205 in keloid fibroblasts, fibroblast cells were transfected with pcDNA, CACNA1G-AS1, si-NC, or si-CACNA1G-AS1. QRT-PCR results determined that the expression of miR-205 was significantly reduced in keloid fibroblasts transfected with CACNA1G-AS1 and was greatly promoted by the knockdown of CACNA1G-AS1 (Figure 4D). In total, miR-205 was lowly expressed in keloids tissues and keloid fibroblasts and miR-205 expression was modulated by CACNA1G-AS1.

### MiR-205 expression influenced the proliferation, invasion, and apoptosis of keloid fibroblasts

**Figure 5:**
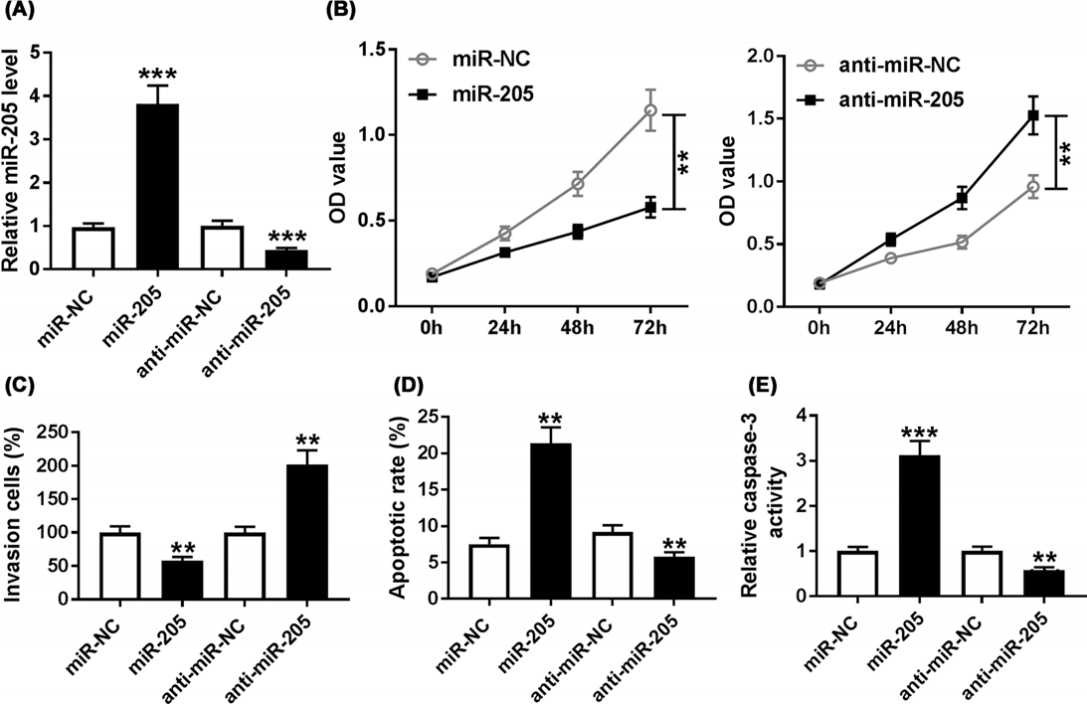
MiR-205 suppressed proliferation and invasion and promoted apoptosis in keloid fibroblasts Keloid fibroblasts were transfected with miR-NC, miR-205, anti-miR-NC, or anti-miR-205, respectively. (**A**) The expression level of miR-205 in transfected keloid fibroblasts was detected by qRT-PCR assay. (**B**) CCK-8 assay was used to evaluate the proliferation of transfected keloid fibroblasts. (**C**) The invasion of transfected keloid fibroblasts was measured by transwell assay. (**D**) Flow cytometry was used to assess apoptosis of transfected keloid fibroblasts. (**E**) The activity of caspase-3 in transfected keloid fibroblasts was examined by Caspase-3 activity assay. **P<0.01, ***P<0.001.

To observe the possible role of miR-205 in keloid fibroblasts proliferation, invasion and apoptosis, keloid fibroblasts were transfected with miR-NC, miR-205, anti-miR-NC, or anti-miR-205, respectively. QRT-PCR assay determined that overexpression of miR-205 triggered a significant promotion in miR-205 expression in keloid fibroblasts, whereas miR-205 expression level was markedly reduced following inhibition of miR-205 (Figure 5A). Moreover, CCK-8 assay demonstrated that the proliferation of miR-205-transfected keloid fibroblasts was significantly inhibited, whereas miR-205 silencing promoted growth of keloid fibroblasts (Figure 5B). Besides, transwell assay implied that silenced miR-205 impeded invasion of keloid fibroblasts, while was enhanced miR-205 overexpression (Figure 5C). Furthermore, flow cytometry analysis showed that the apoptosis rates of keloid fibroblasts transfected with miR-205 were dramatically increased compared with control groups, while the apoptosis rates of keloid fibroblasts transfected with anti-miR-205 were significantly reduced (Figure 5D). In addition, the up-regulation of caspase-3 activity was found by miR-205 transfection, while miR-205 knockdown prominently reduced the activity of caspase-3 compared with control groups in keloid fibroblasts (Figure 5E). These data indicated that miR-205 was involved in the proliferation, invasion, and apoptosis of keloid fibroblasts *in vitro*.

### CACNA1G-AS1 modulated proliferation, invasion, and apoptosis of keloid fibroblasts by binding miR-205

**Figure 6:**
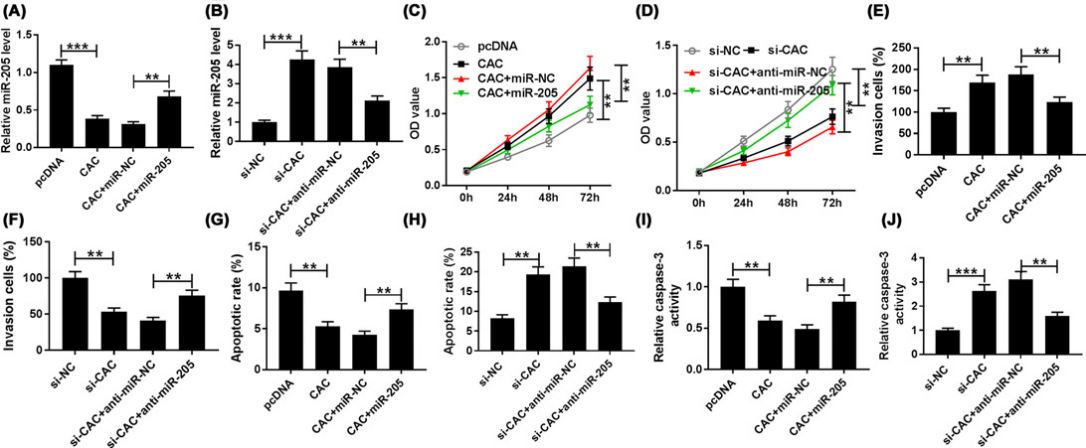
CACNA1G-AS1 down-expression reversed effects caused by miR-205 knockdown on proliferation, invasion, and apoptosis of keloid fibroblasts Keloid fibroblasts were transfected with pcDNA, CACNA1G-AS1, CACNA1G-AS1 + miR-NC, CACNA1G-AS1 + miR-205, si-NC, si-CACNA1G-AS1, si-CACNA1G-AS1 + anti-miR-NC, or si-CACNA1G-AS1 + anti-miR-205. (**A,B**) The expression level of miR-205 was measured in transfected keloid fibroblasts by qRT-PCR assay. (**C,D**) Cell proliferation of transfected keloid fibroblasts was assessed by CCK-8 assay. (**E,F**) Cell invasion of transfected keloid fibroblasts was detected by transwell assay. (**G,H**) Flow cytometry was performed to measure apoptosis of transfected keloid fibroblasts. (**I,J**) The activity of caspase-3 in transfected keloid fibroblasts was measured by Caspase-3 activity assay. **P<0.01, ***P<0.001.

To make clear the function of CACNA1G-AS1/miR-205 axis in keloid fibroblasts, we measured the expression level of miR-205 in keloid fibroblasts. Compared with the negative control, the level of miR-205 was conspicuously reduced by the overexpression of CACNA1G-AS1 and apparently promoted when keloid fibroblasts were co-transfected with miR-205 (Figure 6A). Similarly, miR-205 level was increased by the knockdown of CACNA1G-AS1, however, was then decreased by transfection with anti-miR-205 (Figure 6B). CCK-8 assay determined that the proliferation of keloid fibroblasts was promoted by the overexpression of CACNA1G-AS1, when co-transfection with miR-205, the proliferation was significantly reduced in keloid fibroblasts (Figure 6C). Meanwhile, the proliferation of keloid fibroblasts was greatly impeded by the interference of CACNA1G-AS1 compared with the negative control, which was significantly promoted following the transfection with anti-miR-205 (Figure 6D). Also, transwell assay indicated that the overexpression of CACNA1G-AS1 promoted the invasion of keloid fibroblasts which was then decreased by co-transfection with miR-205 (Figure 6E). Meantime, the invasion of keloid fibroblasts was largely blocked by CACNA1G-AS1 silencing, while the invasion of keloid fibroblasts was significant increased when transfected with si-CAC + anti-miR-205 (Figure 6F). Moreover, the apoptosis of keloid fibroblasts was dramatically inhibited in keloid fibroblasts transfected with CACNA1G-AS1 compared with control groups and was then enhanced by the introduction with miR-205 (Figure 6G). The apoptosis was markedly promoted in keloid fibroblasts transfected with si-CACNA1G-AS1, which was reversed by miR-205 down-regulation (Figure 6H). The activity of caspase-3 was measured in transfected keloid fibroblasts. The results showed that caspase-3 activity was promoted by silenced CACNA1G-AS1 and was then inhibited when co-transfection with anti-miR-205, which further confirmed the interaction between CACNA1G-AS1 and miR-205 in apoptosis of keloid fibroblasts (Figure 6I,J). These findings implied that CACNA1G-AS1 regulated proliferation, invasion and apoptosis of keloid fibroblasts through suppressing miR-205 expression.

## Discussion

Keloid is caused by aberrant proliferation of the skin [23]. Keloid brings a series of functional problems to the health, such as: itching and pain [24]. It has been surveyed that more than 11 million people were affected with keloids worldwide [25]. However, the high recurrence rate is still the biggest problem up to now. Emerging studies showed that the promotion of apoptosis and inhibition of proliferation in keloid fibroblasts facilitated to improve the keloid formation [26]. In the present study, we illuminated the role of CACNA1G-AS1 and the molecular mechanism between CACNA1G-AS1 and miR-205 in human keloid fibroblasts.

CACNA1G-AS1 has been proved to associate with progression in multiple diseases, such as hepatocellular carcinoma, non-small cell lung cancer (NSCLC) and keloid. The previous study demonstrated that the CACNA1G-AS1 expression was greatly increased in keloid relative to normal skin [11]. Li et al. reported that CACNA1G-AS1 promoted the cell migration of keloid fibrosis [27]. Consistent with above studies, we discovered that CACNA1G-AS1 level was significantly increased in keloid tissues and keloid fibroblasts. CACNA1G-AS1 overexpression and knockdown exerted the opposite roles in proliferation, invasion and apoptosis of keloid fibroblasts, suggesting that CACNA1G-AS1 was closely related to progression of keloid fibroblasts.

MiR-205 has been widely reported to act as a suppressor in many diseases. MiR-205-5p expression was reduced in pulmonary arterial hypertension and miR-205-5p suppressed the proliferation of pulmonary vascular smooth muscle by binding MICAL2 mRNA [28]. Also, miR-205 could down-regulate HOXD9 expression to suppress EMT and tumor development [29]. In keloid, An et al. demonstrated that miR-205-5p up-regulation noticeably hindered cell viability, triggered cell apoptosis, and blocked cell invasion and migration ability in human keloid fibroblasts [22]. Consistent with the results of An et al., the level of miR-205 was greatly reduced in keloid tissues and keloid fibroblasts, and miR-205 suppressed proliferation and invasion and promoted apoptosis of keloid fibroblasts. These data proved that miR-205 acted as an inhibitory factor to suppress cell progression of keloid fibroblasts.

Interestingly, miR-205 expression was oppositely correlated with CACNA1G-AS1 expression. Moreover, miR-205 level was down-regulated by CACNA1G-AS1. Dual luciferase reporter and RIP assays also confirmed the directly interaction between CACNA1G-AS1 and miR-205. Furthermore, we discovered that CACNA1G-AS1 and miR-205 had the opposite function in proliferation, invasion, and apoptosis of keloid fibroblasts. These above data implied that CACNA1G-AS1/miR-205 axis regulated miR-205 expression to modulate the proliferation, invasion, and apoptosis in keloid fibroblast.

All of the above results revealed that lncRNA CACNA1G-AS1 enhanced proliferation and invasion and inhibited apoptosis by sponging miR-205 in human keloid fibroblasts. The data suggested that CACNA1G-AS1 might be a novel treatment target for keloids. However, all these data were all achieved in keloid fibroblasts *in vitro*, no *in vivo* experiments were performed in the mice model or patients.

## Conclusions

CACNA1G-AS1 was highly expressed in keloid tissues and keloid fibroblasts, and CACNA1G-AS1 regulated proliferation, invasion, and apoptosis in keloid fibroblasts. CACNA1G-AS1 was predicted to interact with miR-205 and miR-205 expression was down-regulated by CACNA1G-AS1. Also, the expression of miR-205 was up-regulated in keloid tissues and keloid fibroblasts, and miR-205 modulated proliferation, invasion, and apoptosis in keloid fibroblasts. Moreover, lncRNA CACNA1G-AS1 suppressed miR-205 expression to promote proliferation and invasion and inhibit apoptosis of human keloid fibroblasts. These findings provided new evidence for the better understanding of CACNA1G-AS1 and a novel potential diagnostic target of keloid.

## Abbreviations

ANOVA: analysis of variance
BCA: bicinchoninic acid
CACNA1G-AS1: calcium voltage-gated channel subunit alpha1 G antisense RNA 1
CCK-8: Cell Counting Kit-8
ceRNAs: competing endogenous RNAs
FITC: fluorescein isothiocyanate
KF: keloid fibroblasts
lncRNAs: long non-coding RNAs
miRNAs: microRNAs
NF: normal fibroblasts
PBS: phosphate-buffered saline
qRT-PCR: quantitative real-time polymerase chain reaction
RIP: RNA immunoprecipitation
siRNA: small interference RNA
